# Poloxam Thermosensitive Hydrogels Loaded with hFGF2-Linked Camelina Lipid Droplets Accelerate Skin Regeneration in Deep Second-Degree Burns

**DOI:** 10.3390/ijms232112716

**Published:** 2022-10-22

**Authors:** Yuan Zhang, Wanying He, Shuhan Zhang, Xingli Hu, Siming Sun, Hongtao Gao, Jie Kong, Hongxiang Liu, Haiyan Li, Xin Liu, Yan Cheng

**Affiliations:** 1 College of Life Science, Engineering Research Center of the Chinese Ministry of Education for Bioreactor and Pharmaceutical Development, Jilin Agricultural University, Changchun 130118, China; 2 College of Tropical Crops, Hainan University, Haikou 570228, China

**Keywords:** camelina lipid droplets, human basic fibroblast growth factor (hFGF2), poloxam hydrogels, burn, skin regeneration

## Abstract

Burn injuries are difficult to manage due to the defect of large skin tissues, leading to major disability or even death. Human fibroblast growth factor 2 (hFGF2) is known to promote burn wound healing. However, direct administration of hFGF2 to the wound area would affect the bioactivity. To provide a supportive environment for hFGF2 and control its release in a steady fashion, in this research, we developed novel thermosensitive poloxam hydrogels delivered with hFGF2-linked Camelina lipid droplets (CLD-hFGF2 hydrogels). Cryopreserved scanning electron microscopy (SEM) results indicated that the incorporation of CLD-hFGF2 does not significantly affect the inner structure of hydrogels. The rheological properties showed that CLD-hFGF2 hydrogels gelated in response to temperature, thus optimizing the delivery method. In vitro, CLD-hFGF2 could be released from hydrogels for 3 days after drug delivery (the release rate was 72%), and the release solution could still promote the proliferation and migration of NIH3T3 cells. In vivo, compared with hydrogels alone or with direct CLD-hFGF2 administration, CLD-hFGF2 hydrogels had the most obvious effect on deep second-degree burn wound healing. This work indicates that CLD-hFGF2 hydrogels have potential application value in burn wound healing.

## 1. Introduction

Burns are a common type of injury in humans caused by exposure to hot liquids or vapors [1,2,3], with approximately 500,000 burn cases registered each year and 40,000 patients hospitalized [4,5,6]. Similar physical features are present in all types of burns, such as wound exudates and necrotizing debris on the surface. Burns involving epidermal and dermal injuries can be clinically classified as superficial second-degree burns, deep second-degree burns, and third-degree burns [7,8]. Medications can only improve the healing process of first-degree burns; second- and third-degree burns require immediate hospitalization [9,10,11]. Due to the lack of dermis, full-thickness skin defects cannot repair themselves autologously. Functional scaffolds must be constructed to provide an extracellular matrix (ECM) analogue and a suitable environment for wound healing. Traditional wound dressings for burn wound management easily adhere to the wound after absorbing the wound exudate and thus easily tear the new tissues, and the wound is re-injured when the dressing is removed [12,13,14]. Therefore, the ideal wound dressing for the treatment of burns should be well adapted to irregular wound shapes, easily applied to a wide range of wound areas, and enhance the therapeutic effect of wound healing drugs.

hFGF2, as a member of the fibroblast growth factor superfamily, is known to promote proliferation, migration, and differentiation of fibroblasts [15]. Through enhancing therapeutic angiogenesis and tissue regeneration, hFGF2 plays an important role in embryonic development and tissue repair [16,17]. Among cytokines, hFGF2 showed endogenous immunolocalization in the human dermis in partial-thickness burns from day 4 to day 11 [18]. In adult second-degree burns, the topical administration of hFGF2 exhibited obvious regeneration of granulation tissues and angiogenesis in a randomized controlled clinical trial within 5 days after injury [19]. However, the presence of proteases and degradation reactions in the wound cause hFGF2 to be easily broken down [20]. The need for repeated administration of hFGF2 in the treatment of wounds brings inconvenience to patients and increases the risk of infection. Therefore, it is challenging to release hFGF2 continuously at the burn site without losing its biological activity and to reduce the frequency of changing dressings and promote wound repair.

Plant lipid droplets (as well as oil bodies, CLD), as spherical suborganelles in oil crop seeds, are internally composed of triacylglycerol and externally wrapped by a monolayer formed by phospholipids and oil globulin and have been applied in drug delivery systems due to their safety [21,22,23,24]. Previously, PCR fusion technology was used to connect the hFGF2 protein to the surface of CLD, and its particle size was reduced to 133.5 nm [25]. Compared with hFGF2, CLD-hFGF2 has a more significant therapeutic effect on wound healing, improves transdermal absorption efficiency, and solves the disadvantage that the hFGF2 protein is unstable and has difficulty penetrating the cuticle of skin. Therefore, it is necessary to develop a dressing suitable for loading lipid droplets which can be used to continuously release CLD-hFGF2 at the wound site to facilitate drug administration while maintaining the structure of lipid droplets. It is necessary to develop a dressing suitable for loading CLD-hFGF2.

Polymer hydrogels are considered the preferred biomaterial for developing burn wound dressings due to their hydrophilic properties and soft tissue similarity [26,27,28,29,30]. Hydrogels have a three-dimensional structure composed of hydrophilic substances, which can be used as promising protective agents to simultaneously load and transport biological macromolecules [31,32]. Hydrogels are insoluble in water, so they can absorb water equal to 10% to thousands of times their weight [33]. Meanwhile, they have sufficient moisturizing ability for the wound and play a positive role in cleaning necrotic tissues [34]. Hydrogels have been used to treat pressure ulcers, surgical wounds, burns, and radioactive dermatitis [35,36]. Poloxam is composed of hydrophilic ethylene oxide (EO) and hydrophobic propylene oxide (PO) blocks, with EO-POB-EOA as the basic structure [37]. Because of its unique sol–gel transition behavior in aqueous solution and temperature response behavior in aqueous solution, it has been widely used as a proprietary drug carrier [38]. These copolymers form spherical micelles in aqueous solution through hydrophobic interactions between intermediate PPO chain segments [39]. When the critical temperature and concentration of gelation are reached, self-assembled micelles are tightly packed to form a gel structure that is physically cross-linked [40]. Due to the absence of a crosslinking agent, poloxam is a potentially suitable drug carrier and has been used to load aFGF in spinal cord injury repair [41], in preventing post-laminectomy epidural adhesion and other postoperative adhesions, and in ophthalmic drug delivery [42,43].

In this research, we investigated whether the combination of poloxam hydrogels and CLD-hFGF2 could accelerate wound healing and reduce the number of times applied while providing better treatment results than CLD containing hFGF2 directly. Therefore, poloxam composite hydrogels were synthesized from P188 and P407. These spherical micelles have polyoxypropylene cores and expanded polyoxyethylene chains as shells. As the temperature rises, the hydrophobic polyoxypropylene segments in the hydrogels lose water and polymerize into micelles. As the temperature continues to rise, the micelles arrange to form hydrogels. They can gel quickly on the surface of burn wounds, reduce the loss of drug flow, and improve the drug utilization rate. Then, the physical properties of the composite hydrogels were characterized by cryogenic scanning electron microscopy and rheometry. The release state and biological activity of lipid droplets were studied by in vitro release and cell proliferation and migration experiments. In addition, compound hydrogels containing hFGF2 Camelina lipid drops were tested for the treatment of deep second-degree burns in mice, and histological observation was conducted to research the application of compound hydrogels containing hFGF2 Camelina lipid drops in the treatment of burns.

## 2. Results and Discussion

### 2.1. Preparation and Characterization of the CLD-hFGF2 Hydrogels

**Figure 1:**
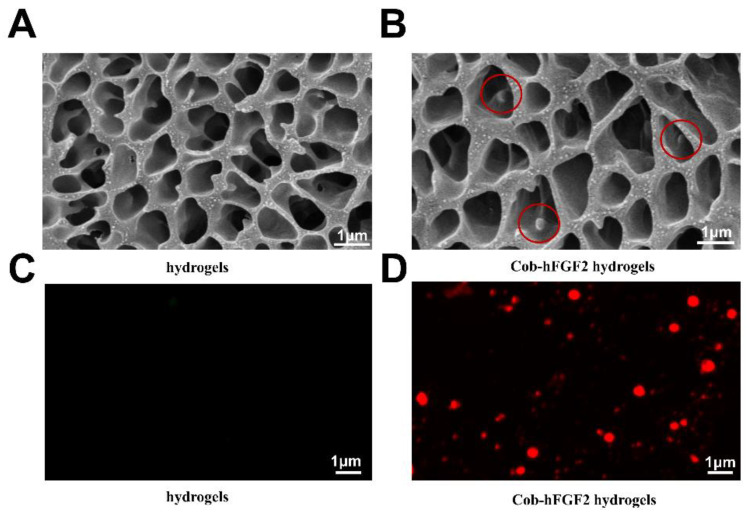
Characterization of CLD-hFGF2 hydrogels. Microstructure of the blank hydrogels (**A**) and CLD-hFGF2 hydrogels (**B**) observed using Cryo-SEM (red circle represents CLD-hFGF2). Fluorescence image of blank hydrogels (**C**) and CLD-hFGF2 hydrogels (**D**) (scale bar = 1 μm, n = 3).

Cryo-SEM was used to characterize the microstructure of the poloxam hydrogels and CLD-hFGF2 hydrogels. Figure 1A shows the loose network structure of the hydrogels, which ensures the successful release of the lipid droplets without damaging the structure after loading. The hydrogels loaded with CLD-hFGF2 were also observed, and the porosity and interconnectivity of the hydrogels remained nearly unchanged and were not affected by loaded CLD-hFGF2. The loaded CLD-hFGF2 can be seen in the holes, uniform spheres with a size of approximately 133.5 nm (Figure 1B red circle) [25]. Confocal microscopy was used to observe the images of Nile Red-labeled CLD-hFGF2 after gelation, containing about 19 CLD-hFGF2 lipid droplets per mm^2^. Most of the dimensions were consistent with the SEM results, except for the appearance of partial aggregation (Figure 1C,D). These results indicate that poloxam hydrogels are suitable for the absorption of CLD-hFGF2.

### 2.2. Injectable Temperature Sensitivity and Rheological Detection of Hydrogels

**Figure 2:**
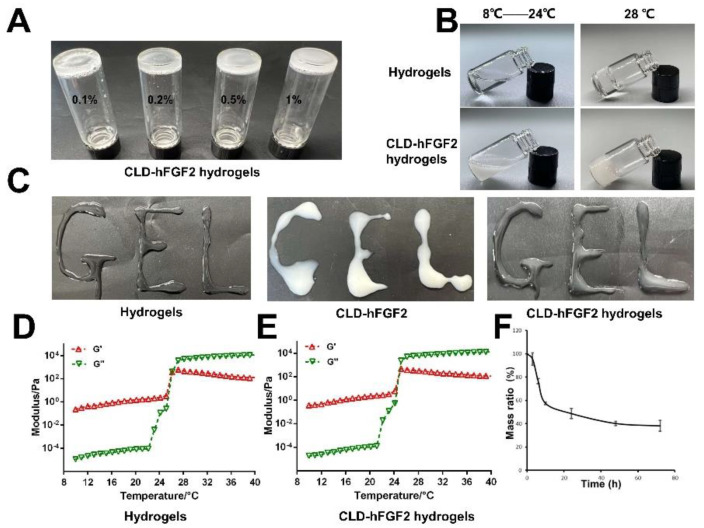
The thermosensitive property of CLD-hFGF2 hydrogels and in vitro degradability. (**A**) Digital photos illustrating hydrogels with different CLD-hFGF2 contents. (**B**) Digital photos illustrating the sol–gel transition of CLD-hFGF2 hydrogels. (**C**) Injectable properties of hydrogels. (**D**) (**E**) Storage modulus (G′), loss modulus (G″) of hydrogels and CLD-hFGF2 hydrogels as functions of temperature. (**F**) Degradation of the CLD-hFGF2 hydrogel in vitro.

The CLD-hFGF2 contents were 0.1%, 0.2%, 0.3%, 0.5%, and 1% hydrogels. The hydrogels with different CLD-hFGF2 contents showed different transparencies, but all of them could gelatinize in response to temperature (Figure 2A). The states of hydrogels before and after loading CLD-hFGF2 at different temperatures are shown in Figure 2B. The hydrogels were flowable at 8 °C, and gelation gradually occurred at 22 °C and became a solid hydrogel at 28 °C. CLD-hFGF2 was uniformly dispersed in the hydrogels, and hydrogel formation was not affected. When the temperature dropped to 8 °C, the hydrogels became liquid again and could be reformed into hydrogels (Figure 2B). Hydrogels were somewhat injectable, and CLD-hFGF2 alone cannot form letters. Hydrogels can easily write gelatinous letters, a property that did not change with the addition of CLD-hFGF2 (Figure 2C).

The gelation temperature before (Figure 2D) and after (Figure 2E) the addition of CLD-hFGF2 was detected by a rheometer. In this experiment, two gelatinous parameters, namely storage modulus (G′) and loss modulus (G″), reflect the change in viscosity and elasticity, respectively. The G′ and G″ values of hydrogels increased quickly at temperatures between 25 and 27 °C, indicating a process of sol–gel phase transition (Figure 2D). Thus, CLD-hFGF2 can easily form hydrogels at body temperature (37 °C). In addition, the phase transition temperature of CLD-hFGF2 hydrogels decreased to 24–25 °C, which was a slight difference from hydrogels alone (Figure 2E). This may be due to micelle packing and entanglements of poloxamer 407 and P188 being disturbed after incorporating CLD-hFGF2. The results indicate that CLD-hFGF2 hydrogels have a phase transition temperature suitable for drug loading and wound administration.

Effective wound dressings have a certain degree of degradability. For this purpose, we evaluated the degradability of hydrogels in vitro (Figure 2F). The experiment lasted for 72 h, and the weight of the hydrogels decreased to approximately 40%, proving that it had certain self-degradation level, and the remaining gel could continue to release the drug loaded.

### 2.3. Controlled Release Profile and Cell Proliferation

**Figure 3:**
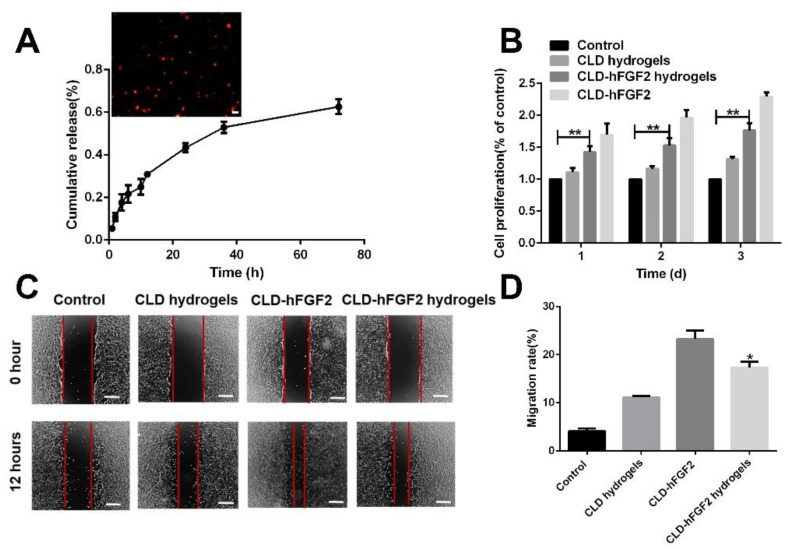
Cell proliferation and migration activity of CLD-hFGF2 released by hydrogels. (**A**) The CLD-hFGF2 release profiles from the hydrogels and the shape of the released CLD-hFGF2. (**B**) Fibroblast cell proliferation ability under PBS treatment. CLD was released from hydrogels, and CLD-hFGF2 and CLD-hFGF2 were released from hydrogels. (**C**) Fibroblast cell migration ability and rate of cell migration under PBS, CLD released from hydrogels, CLD-hFGF2, CLD-hFGF2 released from hydrogels. (**D**) Rate of cell migration under PBS, CLD released from hydrogels, CLD-hFGF2, CLD-hFGF2 released from hydrogels (n = 3; ** *p* < 0.01; scale bar = 50 μm).

The controlled release profile of CLD-hFGF2 from the CLD-hFGF2 hydrogels were obtained by ELISA (Figure 3A). CLD-hFGF2 released from hydrogels showed an initial burst release of approximately 30% after 12 h, followed by a slow release over 72 h, with almost 60% of CLD-hFGF2 being released by hour 72. After 72 h, the released solution was collected and stained with Nile red dye for observation. CLD-hFGF2 was completely shaped and dispersed (Figure 3A). Then, the CLD-hFGF2 released from the CLD-hFGF2 hydrogels was taken on days 1, 2, and 3 and added to NIH/3T3 cells that had been cultured in a 96-well plate for one day, and CLD-hFGF2 was tested by cell proliferation studies (Figure 3B). Compared with the untreated control group, the solution released from CLD-hFGF2 hydrogels for different days significantly promoted the cell proliferation of CLD-hFGF2 (Figure 3B), and CLD-hFGF2 without hydrogel loading was used as the positive control group.

CLD-hFGF2 released at 24 h was collected for the cell scratch test. As shown in Figure 3C, compared with the PBS group, CLD-hFGF2 released from hydrogels promoted cell migration. Compared with the CLD-hFGF2 group, the cell migration rate was lower in the CLD-hFGF2 hydrogel group because the released concentration of CLD-hFGF2 from hydrogels at 24 h was less than that of CLD-hFGF2 treatment alone. CLD-hFGF2 is one of the main components of fibroblast proliferation in the early stage of wound healing, and promoting the proliferation of fibroblasts plays a crucial role in promoting wound healing. The proliferation of fibroblasts was continuously promoted by CLD-hFGF2 loaded on hydrogels, which may be due to the relatively loose network of non-crosslinked hydrogels, thus releasing CLD-hFGF2 continuously. The sustained release of CLD-hFGF2 promoted cell migration, and NIH/3T3 cell migration is necessary to promote wound healing. These results indicate that CLD-hFGF2 can be released continuously from the hydrogels and maintain sufficient biological activity. The combination of sustained release and therapeutic effect was more conducive to wound healing.

### 2.4. In Vivo Wound Healing Effect on Mice with Deep Second-Degree Burns

**Figure 4:**
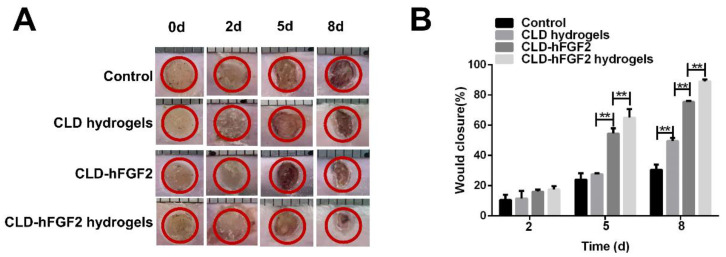
CLD-hFGF2 hydrogels accelerate deep second-degree burn wound healing. (**A**) Images of skin wounds after being covered with PBS, CLD hydrogels, CLD-hFGF2, and CLD-hFGF2 hydrogels. (**B**) Wound healing rates on the 0th, 2nd, 5th, and 8th days (n = 6, ** *p* < 0.01).

To determine the burn healing effect of CLD-hFGF2 hydrogels in vivo, a model of deep second-degree burns in Balb/c mice was established, and the prepared samples were applied to the wound. As shown in Figure 4A, images of burn wounds were taken at different time intervals after treatment in the control group, Cob hydrogel group (as blank hydrogel group), CLD-hFGF2 group (positive group), and CLD-hFGF2 hydrogel group. The results showed that in all treatment groups, the growth of new epidermis extended to the center of the wound, resulting in a reduction in the wound area. Significantly, compared with other groups, the change in wound shrinkage rate after CLD-hFGF2 hydrogel treatment was the fastest on day 5 and day 8 (Figure 4B).

On day 5, the healing rate of the CLD-hFGF2 hydrogel group reached 69%, and treatment with CLD-hFGF2 alone also showed a relatively high healing rate (52%) compared with that of the control group (27%). On day 8, the CLD-hFGF2 hydrogel treatment group was close to complete healing (90%). This was mainly because the hydrogels formed a relatively moist and closed environment at the wound site, which was more conducive to wound healing [34]. On the other hand, the hydrogels prolonged the residence time of CLD-hFGF2 in the wound site and sustained the release of CLD-hFGF2, which further accelerated wound healing. In addition, the CLD hydrogel group had a healing effect compared with the control group, which may be caused by the synergistic result of the anti-inflammatory effect of unsaturated fatty acids in CLD [44] and the wet compress environment of hydrogels. Overall, the above data indicate that loading CLD-hFGF2 on hydrogels significantly improves the therapeutic effect of CLD-hFGF2 and accelerates the wound healing of burns.

### 2.5. Histochemical Analysis of Skin

**Figure 5:**
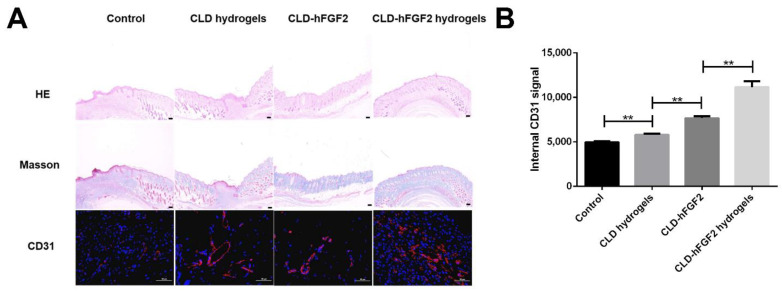
Granulation tissue, collagen, and expression of CD31 for different groups on the wound healing on the 8th day. (**A**) Images of HE, Masson and CD31 after covering with PBS, CLD hydrogel, CLD-hFGF2 and CLD-hFGF2 hydrogel. (**B**) Visualization of data on CD31 signals. (n = 4, ** *p* < 0.01) (HE and Masson staining are shown at 4 × 10 magnification, scale bar = 50 μm; CD31 staining is shown at 40 × 10 magnification, scale bar = 50 μm).

To further evaluate the therapeutic effect of CLD-hFGF2 hydrogels on deep second-degree burn healing, wound tissues of each group were analyzed by H&E staining, Masson staining, and immunofluorescence to evaluate collagen and granulation tissue regeneration and angiogenesis. As shown in Figure 5A, on day 8, the CLD-hFGF2 hydrogel group and CLD-hFGF2 group showed tighter and more aligned collagen fibers in the extracellular matrix. In contrast, collagen fibers were irregularly arranged and loosely packed in the other groups. Moreover, the tissue treated with CLD-hFGF2 hydrogels had better epithelial regeneration, more developed granulation tissue, and more connective tissue stroma. Treatment with CLD-hFGF2 hydrogels significantly increased granulation tissue density and showed a higher level of regeneration than treatment with CLD-hFGF2 (Figure 5A).

CD31 is an important biomarker of vascular endothelial cells. The immunofluorescence intensity of the angiogenesis-related factor CD31 is shown in Figure 5A,B. In the CLD-hFGF2 hydrogel treatment group, neovascularization in the subcutaneous tissue around the wound was the most obvious. These results suggest that CLD-hFGF2 hydrogels accelerate the re-epithelialization of the burn wound and promote collagen deposition and angiogenesis. The sustained release of CLD-hFGF2 from the poloxam hydrogels enhanced ECM remodeling and reconstruction, allowing the collagen fiber arrangement to be close to that of normal tissue.

## 3. Materials and Methods

### 3.1. Materials

Poloxamer (P407, P188, Shanghai Houcheng Fine Chemical Co., Ltd., Shanghai, China), polyethylene glycol (PEG, relative molecular weight: 400, Tianjin Guangfu Fine Chemical Research Institute), polyvinyl alcohol (PVA 0388, Colaoli Co., Ltd., Tokyo, Japan), and glycerol (Tianjin Guangfu Fine Chemical Research Institute, Tianjin, China) were used. The ELISA kit (hFGF2) was purchased from Baijin Biological (Beijing Soleibao Biology Co., Ltd., Beijing, China). Transgenic Camelina seeds were cultivated and stored by Jilin Agricultural University. All solvents and reagents utilized in this research were of analytical grade.

### 3.2. Preparation of CLD-hFGF2 Hydrogels

Hydrogels were synthesized by poloxam (P407 and P188) and PVA. First, P407 and P188 were dissolved in 0 °C water and stirred until they fully dissolved while glycerin was added. The transparent solution was obtained after overnight storage at 4 °C. Then, the PVA was dissolved in 95 °C water, and PEG was added and stirred overnight at 4 °C. The poloxam solution and PVA solution in the above configuration were stirred at 4 °C until they were evenly mixed, then placed in a closed bottle and irradiated by linear electron accelerator irradiation cross-linking (DZz-2/1) (Changchun Jiyuan Biotechnology Co., Ltd., Changchun, China). Finally, CLD-hFGF2 was added and mixed well (extraction method of CLD) [25].

### 3.3. Cryo-SEM and Fluorescence Images of CLD-hFGF2 Hydrogels

To investigate the microstructure of hydrogels, ZEISS Sigma 300 (Added freezing module) was utilized to scan the samples. Cryo-SEM was used in this examination. Cryogenic preparation does not destroy the structure of the sample, and it can better observe the state of CLD-hFGF2 in hydrogels. The dehydrated specimens were cross-sectioned and sputter-coated with gold, followed by scanning observation (n = 3). Laser confocal microscopy (Leica TCS-SP8 SR) was used to observe CLD-hFGF2 hydrogels after Nile Red labeling.

### 3.4. Rheological Detection of CLD-hFGF2 Hydrogels

Hydrogels and CLD-hFGF2 hydrogels were measured by a rheometer (Antonpah MCR702, Australia) at different temperatures ranging from 10 to 40 °C [45]. Stainless steel parallel plates (25 mm) were used to measure elastic and viscous shear moduli and draw rheological curves.

### 3.5. In Vitro Release of CLD-hFGF2 Hydrogels and Degradability

Enzyme-linked immunosorbent assay (ELISA) was used to detect the release kinetics of CLD-hFGF2 from hydrogels [46]. Hydrogels containing CLD-hFGF2 were first soaked in pH 7.4 PBS and placed on a shaker at 37 °C. At 1, 2, 3, 4, 6, 8, 10, 12, 24, 36, and 72 h, 1 mL of the PBS solution was obtained and stored at −20 °C for the final analysis [41]. Meanwhile, 1 mL of fresh PBS was added to the system. The release kinetics of CLD-hFGF2 from the hydrogels were obtained by ELISA following the manufacturer’s protocol. In total, 3 days of cumulative release were recorded for the CLD-hFGF2 hydrogels with CLD-hFGF2 samples. The cumulative release percentage (%) was calculated by the following formula: cumulative release percentage = (amount in supernatant/the initial amount of CLD-hFGF2 in the hydrogels) × 100%.

The hydrogels were placed in PBS solution and incubated for 72 h, and the hydrogel weights were measured at different time points. The degradation behavior of hydrogels in PBS solution for 72 h was measured. The degradation rate (De) was calculated according to the equation De = (W0 − Wt)/W0, where W0 is the initial weight of the hydrogels and Wt is the weight of the freeze-dried sample measured at different time points [47].

### 3.6. Cell Proliferation of CLD-hFGF2 Hydrogels

The cells were cultured in 96-well plates using Dulbecco’s modified eagle medium (DMEM) and 1% penicillin/streptomycin antibiotic solution containing 10% FBS for 24 h [48]. CLD-hFGF2 released from hydrogels was divided into a 96-well plate and sterilized by UV light for 1 h. Then, 5000 NIH/3T3 cells were seeded into the wells. Fibroblast cells were cultured and divided into four groups: CLD-hFGF2 (released from hydrogels at 12, 24, and 72 h) as the sample group, CLD-hFGF2 (100 ng/mL) as the positive group, CLD (released from hydrogels at 12, 24, and 72 h) as the negative group, and PBS (pH = 7.4, 0.01 M; 100 µL) as a blank group.

### 3.7. In Vitro Wound Assay

The migration of NIH/3T3 cells was analyzed by standard wound healing scratch assay. After being cultured in Dulbecco’s modified eagle medium (DMEM) containing 10% fetal bovine serum for 24 h, a linear scratch of 1 mm was made on a 6-well plate with an aseptic transfer head. The cells were washed with PBS to remove debris. Fibroblasts were cultured and divided into 4 groups: CLD-hFGF2 (released from hydrogels over 72 h) as the sample group, CLD-hFGF2 (100 ng/mL) as the positive group, CLD (release from hydrogels over 72 h) as the negative group, and PBS (pH = 7.4, 0.01 M; 100 µL) as a blank group. The cell images were observed and collected by an Olympus IX70 microscope at 0 h and 12 h. The scratch closure rate of fibroblasts (%) was calculated as follows: scratch wound rate (%) = (X0 − Xt)/X0 × 100%, where Xt is the scratch distance for 12 h and X0 is the initial position [48].

### 3.8. Deep Second-Degree Burn Model In Vivo

Thirty male Balb/c mice (6–8 weeks) were purchased from Yisi Biotechnology Limited Company (Changchun, China). Balb/c mice (n = 30) were divided into four groups: PBS, CLD hydrogels, CLD-hFGF2, and CLD-hFGF2 hydrogels (the content of hFGF2 was 600 ng in 30 µL) (n = 6). The entire protocol and experiment were approved by the Laboratory Animal Resources Department of the Ethics Committee of Jilin Agricultural University. All animals were housed in standard cages with food and water available ad libitum in a specific pathogen-free facility. Animals were allowed to acclimatize for one week before experimentation. According to the standard procedure described in the literature, a mouse model of deep second-degree burns was established. BALB/c mice were first anesthetized by intraperitoneal injection of 1% sodium pentobarbital (0.005 mL g^−1^), their back hair was shaved, and the skin was disinfected with ethanol. Then, a 160 °C preheating rod was applied to the back of each mouse for 10 s, and two burns with a diameter of 5 mm were caused [7]. All mice were housed individually to prevent additional effects on the wound area. In the wound closure research, the changes in wound area were recorded and photographed on the 2nd, 5th, and 8th days. After anesthesia, the wound was removed for histological analysis [41]. The wound closure rate was calculated using the following formula: wound healing rate = (final wound area at 2, 5, and 8 days/initial wound area) × 100% [49].

### 3.9. Histopathological Analysis

After 8 days, the skin tissues of the mice were collected and soaked in 4% paraformaldehyde. The skin was cut into 0.5 μm thick sections, and intact tissues were selected for H&E staining and Masson staining to observe wound healing. CD31 can be used as a marker of vascular endothelial cells, and the expression of CD31 was observed by immunohistochemistry with a fluorescence inverted microscope (OLYMPUS IX51, Tokyo, Japan) [50].

### 3.10. Statistical Analysis

Data are presented as the mean and standard deviation of at least three independent experiments. Statistical analyses were performed using GraphPad Prism 6.01 (GraphPad Software Inc., San Diego, CA, USA). ImageJ was used for statistical and quantitative analyses. Comparisons between two groups or among three groups were conducted by the t test or one-way ANOVA, respectively, and *p* < 0.05 and *p* < 0.01 were considered significant (* *p* < 0.05 and ** *p* < 0.01).

## 4. Conclusions

In this study, we focused on developing a convenient moist dressing to control the release of CLD-hFGF2 to the wound skin. With a proper formula, a composite thermosensitive hydrogel using poloxamer P188 and P407 crosslinked by linear electron accelerator irradiation was successfully prepared. By encapsulating CLD-hFGF2 in thermosensitive hydrogels, the hydrogel substrate created burst-free and sustained release kinetics of CLD-hFGF2. The CLD-hFGF2 released from hydrogels showed high bioactivity in terms of promoting cell proliferation and migration. The in vivo evaluations also indicated that CLD-hFGF2 hydrogels significantly improved burn wound recovery rates, with more rapid regeneration of ECM and angiogenesis. Our results provide a strategy to accelerate the structural and functional recovery of burn wounds. In future studies, the formula will be further optimized for better drug permeation and retention.

## Data Availability

Not applicable.
